# Delivering PACE++ curriculum in community settings: Impact of TARA intervention on gender attitudes and dietary practices among adolescent girls in Bihar, India

**DOI:** 10.1371/journal.pone.0293941

**Published:** 2023-11-03

**Authors:** Sudipta Mondal, William Joe, Santosh Akhauri, Irina Sinha, Putul Thakur, Vikas Kumar, Tushar Kumar, Narottam Pradhan, Abhishek Kumar

**Affiliations:** 1 Monitoring Learning and Evaluation, Project Concern International, Bihar, India; 2 Institute of Economic Growth, Delhi, India; 3 Central University of Gujarat, Gujarat, India; Indian Institute of Dalit Studies (IIDS), INDIA

## Abstract

Adolescence phase has high intrinsic and instrumental relevance. The Transformative Action for Rural Adolescents intervention delivered PACE++ curriculum with innovations to introduce a) health and nutrition sessions and b) delivery of the content in community settings of rural Bihar. This paper examines impact of the intervention showcasing establishment of intergenerational community connect for empowering and invigorating adolescent girls on gender attitude, empowerment and adolescent health and nutrition. The impact evaluation is based on a two-arm (intervention and comparison groups) cluster randomized controlled design with two rounds of representative cross-sectional surveys. The baseline and endline sample comprised of 2327 and 2033 adolescent girls (15–19 years), respectively. Descriptive statistical, difference-in-differences and propensity score matching methods are used to confirm the program impact. The DID and PSM analyses confirm high significance of impact on gender equity norms, diets and nutritional knowledge and understanding of employee related rights and responsibilities. School-going adolescent girls performed better than those who have discontinued formal education. The intervention showcases the importance of delivering the modified PACE curriculum in rural settings through leveraging community platforms. The findings call for greater policy attention on scaling up of similar initiatives for empowerment and social capital development of adolescent girls.

## 1. Introduction

Unequal opportunities and constricting sociocultural norms are inimical to gender equity and development. Curbs and controls at household and community level often disallow women to pursuit their goals for a healthy and dignified living [1, 2]. These socio-cultural norms are implicit rules within communities that dictate how individuals should behave and what is considered the norm. Adolescence is a critical period when gender discrimination and rigid social norms begin to have significant impact on both boys and girls, with potentially grave consequences for girls. Depending on one’s gender, societal value and expectations differ, resulting in harmful practices like child marriage for girls and risky labor for boys among children, adolescents, and young adults. The United Nations Sustainable Development Goals (SDG) recognize these concerns whereby Goal 5 specifically pledges global commitments for achieving gender equality and empowering all women and girls by 2030 [3]. The SDG agenda acknowledges that lacking freedom for social and economic participation coupled with frigid environment for public provisioning such as health and education are longstanding barriers in gender equity [4].

Boosting investments in social sector (mainly health and education) and physical infrastructure such as roads, information technology, water and sanitation etc. are important for increasing female participation in economic activities [5, 6]. These steps are of instrumental relevance yet these are more likely to augment the “hard skills” such as qualifications, mobility and ability-to-work whereas equal attention is necessary to enhance a range of “soft skills” including empowerment, decision-making, communication and planning skills [7]. The latter is increasingly recognized in labor economics as critical gender-sensitive element for personal and professional growth including inculcation of leadership, managerial and entrepreneurial abilities among women [8]. Besides, focus on such skill development through group forums can be also an important means for augmenting social capital among women and girls that is instrumental for health and socioeconomic well-being [9, 10].

In view of such intricacies, various national and international organizations are piloting alternative strategies to promote gender equity and empowerment across socio-economically diverse and resource-poor settings [11]. Some of these interventions are specifically designed to enhance self-confidence and leadership among adolescent girls through school- or work-site based outreach [12]. The theory of change underlying these efforts is derived from the discourse on positive youth development (PYD) that calls for leveraging elements of human behavior, inter-individual relations and their interaction with the developmental systems for overall well-being of youth and adolescents [13, 14]. The PYD framework hypothesizes that developmental assets (i.e., social and ecological elements for growth of health youth including community-based programs) can favorably influence the five Cs viz. competence, confidence, connection, character, and caring among the participants [15]. The 4-H program in the United States has been instrumental in validating the efficacy of the PYD framework and prompted similar interventions in other countries [16].

Notable is the case of the Personal Advancement & Career Enhancement (P.A.C.E.) curriculum designed by Gap Inc. in 2007 –a widely referred PYD model across settings [17]. P.A.C.E curriculum draws upon the science of adolescent development and aims to boost positive identity of youth and adolescents by designing transformative activities for agency and empowerment. The implementation is contextualized and delivered through a group-based participatory approach for wider engagement and sustainability. The PACE curriculum is usually implemented at factory or work-site settings in Asian countries (Bangladesh, Cambodia, China, India, Sri Lanka and Vietnam) [18]. It is also implemented for school-going adolescents through initiatives such as Planning Ahead for Girls’ Empowerment and Employability (PAGE) and Plan-It Girls of the International Centre for Research on Women (ICRW) [19].

The PACE curriculum, however, offers considerable scope for adaptations to meet the wide range of skill development requirements for youth and adolescents in developing countries [20]. Project Concern International (PCI), therefore, strategically advanced the PACE curriculum with two-fold objectives: a) testing efficacy of PACE curriculum in community settings by facilitating linkages with established platforms such as the self-help groups (SHGs) and b) supplementing the PACE curriculum with health and nutrition modules as integrating these is necessary for boosting adolescent life skills. This twin augmentation is referred to as the PACE++ curriculum. PCI launched the Transformative Action for Rural Adolescents (TARA) intervention for delivering the PACE++ curriculum in rural settings of Rohtas district in Bihar, India. The specific aim of the pilot was to test the hitherto unexplored potential of skill and knowledge development initiatives for informing and empowering adolescent girls through rural community platforms.

The intervention was piloted during 2019–21. As part of the initiative, adolescent girls were mobilized in small groups and were trained on various life skills in domains related to women empowerment, employability skills, and adolescent health and nutrition. The pilot focused on strengthening self-reliance of vulnerable adolescent girls so as to enable them to enter the workforce with strong life skills. Against this background, this paper specifically evaluates the impact of the TARA initiative on improving indicators related to self-efficacy and empowerment of adolescent girls for realizing their goals and aspirations. The analyses are based on data from a two-arm cluster randomized controlled design. Before proceeding with the analysis, a brief note on the TARA initiative is in order.

### 2. TARA pilot

Transformative Action for Rural Adolescents (TARA) pilot is an 18-month intervention launched in November 2019 in Rohtas district of Bihar with funding support from Bill and Melinda Gates Foundation (BMGF) and GAP Inc. PCI in coordination with ICRW and Gap Inc. customized the PACE curriculum for the intervention to be consistent with local context and needs of the focus group. The initial contextualizing of PACE++ curriculum and training material on life skills including gender and empowerment, self-efficacy, employability, entrepreneurship, and health and nutrition was done by PCI. The process entailed redesigning of the modules in a simple and easy way to understand *Hindi* with minor modification of games and methodologies to suit project implementation in Bihar. The core capacities of PCI in domains of family planning, women’s health and nutrition were instrumental in this process. Also, the modules were adapted for a community-based outreach format. It is worth noting that the PACE curriculum is essentially designed for schools whereas the TARA project aimed at engagement with adolescent girls in community settings. The PACE curriculum in India was previously administered in school settings but absenteeism and time-constraints in school-based engagement required a community centric approach for delivery of PACE model for wider outreach among adolescents. Accordingly, the 16 sessions under the PACE modules (divided into five categories viz. self-esteem, self-efficacy, resourcefulness, employability, and adolescent health and nutrition) were tailored and contextualized for the target group. The pilot also relied on field visits and interactions to ease the curriculum adoption participatory process for involving adolescent girls. This helped in improving the language and comprehension necessary for elaborating the concepts used in the tools and also aided in conducting effective training sessions with adolescent groups.

The TARA pilot was implemented in two selected blocks of Rohtas district in Bihar. The pilot mobilized a total of 1205 adolescent girls (initially organized in 47 adolescent groups) and trained them on various aspects such as empowerment, employability, adolescent health and nutrition. The average attendance of adolescents in the PACE++ sessions was 86%. Prior to community roll-out, the training facilitators and supervisors also went through an induction training for improving their understanding about the project and also their ability to deliver the PACE++ curriculum. The training involved both classroom teaching (theory and practice) as well as field-based learning for comprehending session delivery in community settings. The introductory module relied on description of the core concept and encouraged discussion on the subject through use of videos on stories and stereotypes. This served as ice-breaking sessions to allow greater interaction between the adolescent girls and the community facilitators. For mobilization purposes, the PCI team made household visits for rapport building and information sharing about the TARA program. Each module was delivered only after trainings of the community facilitators which included theory sessions, group practice sessions and also field based piloting and learning for appropriate delivery of the sessions. The sessions were primarily conducted during the weekends at convenient timings to facilitate greater participation. Overall, the intervention aimed at 100 hours of direct and indirect contact with the adolescents through module sessions, club activities including sharing of information and materials on the various modules. The monitoring of the intervention was done by the project team at the district and the block level with a quality assurance checklist developed to review the conduct of the session. Feedback to community facilitators was also provided based on the monitoring to enhance efficiency of the facilitators and consequently improve training outcomes.

The village-level organizations (VO) and self-help group (SHG) forums (JEEViKA groups under the State Rural Livelihood Mission (SRLM) in Bihar) were also involved in this initiative. Given the vast network of SRLM, there was a high probability of engaging adolescents through active engagement with the SRLM network. In particular, the JEEViKA staff and cadres were oriented along with village organizations referred to as the Social Action Committee (SAC) regarding the rationale and objectives of the TARA project. The self-help group members as part of the JEEViKA were sensitized for gender equity and women empowerment concerns to motivate greater participation of adolescents from their household and neighborhood. The out-of-school adolescents were also approached through *Sakhi Saheli* (friends) forums to boost participation. This was further instrumental as adolescent girls were encouraged to join the TARA club that sought greater linkages with the SHG groups under JEEViKA initiative of the state government. The TARA program also did a mapping of the skill learning preferences of adolescents and all adults were accordingly linked to Rural Self-Employment Training Institute (RSETI) of the Government of India as well as other skill development centers.

Nonetheless, it is worth mentioning that the TARA pilot was not devoid of the disruptions caused due to the COVID-19 pandemic. This affected the planned activities and necessitated some re-designing of the project strategy to connect with the adolescent groups. In particular, the size of the adolescent groups was reduced from 20–25 members to 10–15 members per group. This implied requirements of additional time and human resources to ensure delivery of the adapted PACE curriculum. Also, there was continuous effort to provide support and motivation, through online channels, to enable the adolescent groups to face the challenges posed by the pandemic. PCI initiated several activities (such as drawings, poems, writing essays, motivational stories etc.) for de-stressing and prevention from COVID-19.

With some improvements in COVID-19 situation and lockdown relaxation, the TARA pilot field activities were re-started in October 2020. During the post-COVID period, participation and leadership abilities among adolescent girls was encouraged through celebration of international girl child day (11^th^ day of October), international women’s day (8^th^ day of March) and cluster-level events such as “*betiyon ki awaz*” (translated as voice of the daughters). However, in April 2021 the second wave of COVID-19 further affected the program activities. During this period, PCI initiated responsive support to the girls on COVID-19 through alternative mediums and provided appropriate information to the girls on ‘COVID appropriate behavior’ and also motivated them to be messengers for COVID-19 vaccination.

## 3. Data and methods

### 3.1 Data

The impact evaluation is based on the baseline and endline survey data collected by the PCI team. A two-arm (intervention and comparison groups) cluster randomized controlled design was used based on two rounds of representative quantitative cross-sectional surveys with adolescent girls (15–19 years). The baseline survey was conducted during November 2020 to January 2021 whereas the end of the project survey was carried out during January 2022. The sample size for the evaluation is derived based on the assumption of observing a 10-percentage point change in critical indicators between intervention and comparison arms after the intervention with 80% power and 95% confidence of interval. The basic sample size for each arm is calculated to be 385 which further adjusted for the cluster design approach. In particular, 24 clusters per arm is required based on the assumption of a between-cluster variation of 0.25 along with the intent to interview 48 respondents per cluster. Accordingly, a total sample of 2304 adolescent girls (1152 each in intervention and comparison group) for the baseline and endline survey is finalized.

All the inhabited villages (128) under the two selected blocks of the district are included for randomization. However, villages with less than 100 households or with high school are removed before randomization for ensuring adequacy of eligible sample. A total of 12 villages from each Block are randomly assigned to the two arms (intervention and comparison). The sampling frame for adolescent girls was developed based on mapping and listing operations for identifying eligible in the selected villages. The exercise is repeated for endline survey. Further, from each village a sample of 48 adolescent girls is randomly selected for both baseline and endline surveys. The baseline sample comprised of 2327 adolescent girls (1122 and 1205 in comparison and intervention areas, respectively) and endline sample of 2033 adolescent girls (1040 and 993 in comparison and intervention areas, respectively). However, within the intervention area, 618 adolescent girls reported regular participation in TARA project activities and they constitute the final analytical sample for the intervention group.

The survey was conducted by a team of trained field investigators (females) using structured interview schedules that was pre-tested in a different district of the state. The inclusion criteria is as follows: unmarried adolescent girls (15–19 years) who are usual residents of the village and are willing to participate in the survey. Parental consent (both verbal and written) was also sought before approaching the adolescent girls for the interview. The interview schedule collected information on socioeconomic background of the respondents along with information on the core domains related to empowerment, employment, gender equity and health and nutrition. However, the data does not include any information which could reveal the identity of the participant.

### 3.2 Outcome measures

Core to the TARA’s programmatic approach is an evidence-based, gender-integrated life skills training and mentoring program, called Personal Advancement & Career Enhancement (P.A.C.E.) that addresses five core domains. The impact across these five domains of empowerment, employment and adolescent health and nutrition is assessed through the following measures: a) gender equity attitudes, b) diet and nutrition, c) self-esteem, d) self-efficacy and e) employee roles and responsibilities. The items used for each domain measures are available in S1 Table in S1 File. The scales are applied from globally validated methodologies and was pretested prior to the survey. An 18-item gender equitable men (GEM) scale (with five-point ordered response categories) is administered to examine the views and perceptions regarding roles of men and women (boys and girls) in the society [21, 22]. Knowledge of dietary diversity and dietary practice is assessed based on knowledge of food groups and consumption from a minimum of five food groups in the last 24 hours [23]. The indicator is augmented with awareness related to micronutrient deficiencies such as anemia which is a prominent adolescent health concern in India. The self-esteem scale comprises of eight items to comprehend the attitude of the adolescent girls and perception regarding themselves. The self-esteem scale is scored on five-point ordered response categories from strongly disagree to strongly agree [24]. Self-efficacy is examined through a nine-item scale to evaluate aspects such as effective communication, decision making ability and self-confidence [25]. A set of questions on rights and responsibilities of an employee are asked to examine the knowledge and awareness regarding respectful employment and employability [26].

### 3.3 Correlates

Participation of the adolescent girls in the community activities under the TARA intervention is the main explanatory variable (S2 Table in S1 File). The participation of adolescent girls in community activities is influenced by a complex interplay of socio-economic and demographic factors. These factors can either facilitate or hinder their involvement in such activities, and affect the domain scores. Understanding these correlates is essential for designing interventions and policies that promote and support the active engagement of adolescent girls in their communities. Therefore, the impact of participation on the outcome measures is presented using both unadjusted and adjusted analysis using socioeconomic and demographic correlates as follows: Household size (below four members or above), religion (Hindu, Muslim or others), social group (SCST—scheduled caste or tribes, OBC–other backward classes and Others), family type (joint or nuclear), household wealth quintile (using principal component analysis (PCA) based asset index with 29 items for baseline and endline separately), housing type (*pucca* house build from durable materials or not), cooking fuel (clean fuel or not), family income (BPL/APL–below poverty line or above poverty line as per state government food ration cards) occupation (self-employed, salaried, casual laborers or unemployed), maternal education (illiterate, up to primary, secondary and above), paternal education (illiterate, up to primary, secondary and above), currently in school (yes or no).

### 3.4 Statistical and econometric analysis

Descriptive statistics is provided for the outcome measures and the key explanatory variables (Table 1; S1 Table in S1 File). Cronbach’s alpha for scale reliability for the domain items is adequate and reported in S2 Table in S1 File [27, 28]. Cohen’s *δ* is reported to ascertain the program effect size to outline their relevance from a policy and scalability perspective [29, 30]. Cohen’s *δ* is a standardized effect size measure that quantifies the magnitude of the difference between two groups, such as the intervention group and the control group. The policy relevance of Cohen’s d lies in its ability to convey the size of the effect in standardized units which allows us to compare scores across domains. Two types of econometric models are applied to examine the strength of the association between exposure variable (participation in TARA intervention) and its impact on the five outcome measures. First, we create binary variable categories for the domain scores (top two quintile scores vs bottom three quintile scores) and apply logistic regression model to understand the association of program exposure with the likelihood of being in upper quintiles of domain scores. Second, as the five domain scales also resemble the properties of count data, we apply Poisson regression model to understand the change in expected scores that can be associated with program participation. Finally, two prominent econometric approaches for impact evaluation–viz. difference-in-differences (DID) and propensity score matching (PSM)—are applied for confirming the impact of program exposure on the selected domains. The DID method compares the outcome measures of intervention and comparison group across baseline and endline period [31]. The DID coefficient is also adjusted for age of the adolescent, schooling status, maternal and paternal education, household size, social group, poverty status and household assets-based wealth quintile. Since about two-third of the endline participants reported greater program exposure hence, as a sensitivity check, the impact ascertained from PSM analysis is presented to account for any variations in background characteristics across baseline and endline sample [32]. The propensity score is generated based on age of the adolescent, schooling status, maternal and paternal education, household size, social group, family type, housing material, poverty status and household assets-based wealth quintile. The propensity scores are based on usual caliper settings of 0.1 (the maximum distance imposed for which two observations are potential neighbors). Both average treatment effect (ATE) and average treatment effect on the treated (ATET) estimates from the PSM analysis is reported. All the analysis is conducted in Stata 15.0.

**Table 1:** Background characteristics of the sample from control and intervention areas.

Background characteristics	Baseline				Endline			
	Comparison		Intervention		Comparison		Intervention	
	N	%	N	%	N	%	N	%
Age (in years)								
15 years	453	40.4	501	41.6	186	17.9	202	20.3
16 years	211	18.8	226	18.8	254	24.4	200	20.1
17 years	183	16.3	221	18.3	218	21.0	211	21.3
18 years	161	14.3	143	11.9	163	15.7	184	18.5
19 years	114	10.2	114	9.5	219	21.1	196	19.7
Currently in school								
Yes	963	85.8	1047	86.9	832	80.0	809	81.5
No	159	14.2	158	13.1	208	20.0	184	18.5
Mother’s education								
No formal schooling	580	51.7	616	51.1	501	48.2	501	50.5
Up to primary	302	26.9	339	28.1	300	28.8	285	28.7
More than primary	240	21.4	250	20.7	239	23.0	207	20.9
Father’s education								
No formal schooling	287	25.6	322	26.7	258	24.8	257	25.9
Up to primary	251	22.4	260	21.6	228	21.9	202	20.3
More than primary	584	52	623	51.7	554	53.3	534	53.8
Household size								
Up to 4 members	177	15.8	206	17.1	177	17.0	184	18.5
More than 4 members	945	84.2	999	82.9	863	83.0	809	81.5
Social group								
SC/ST	235	20.9	339	28.1	219	21.1	270	27.2
OBC	840	74.9	802	66.6	781	75.1	673	67.8
Others	47	4.2	64	5.3	40	3.8	50	5.0
BPL household								
Yes	688	61.3	753	62.5	653	62.8	645	64.9
No	434	38.7	452	37.5	387	37.2	348	35.1
Household wealth quintile								
Lowest	197	17.6	270	22.4	206	19.8	201	20.2
Second	212	18.9	253	21.0	185	17.8	225	22.7
Middle	233	20.8	232	19.3	211	20.3	192	19.3
Fourth	250	22.3	216	17.9	218	21.0	189	19.0
Highest	230	20.5	234	19.4	220	21.2	186	18.7
All	1122	100.0	1205	100.0	1040	100.0	993	100.0

## 4. Results

The background characteristics of the intervention and comparison group across baseline and endline period is similar (Table 1). Over 80% of the adolescents are currently in school. A large proportion of parents do not have any formal schooling (50% among mothers and 25% among fathers). Almost all the households belong to socially backward groups; one-fourth of them from scheduled caste or tribes (SC/ST) and two-thirds from other backward classes (OBC). Two third-of the households are categorized as poor based on the below poverty line (BPL) food ration card issued by the government. Some variations in the age distribution are noted. In the baseline sample about 40% of the adolescents were 15 years old and in endline sample one-fifth are aged 15 years. This is mainly because the intervention aimed to capture those who were the part of the same age-cohort. The proportion of 19 years old is 10% and 20% in baseline and endline survey.

**Fig 1:**
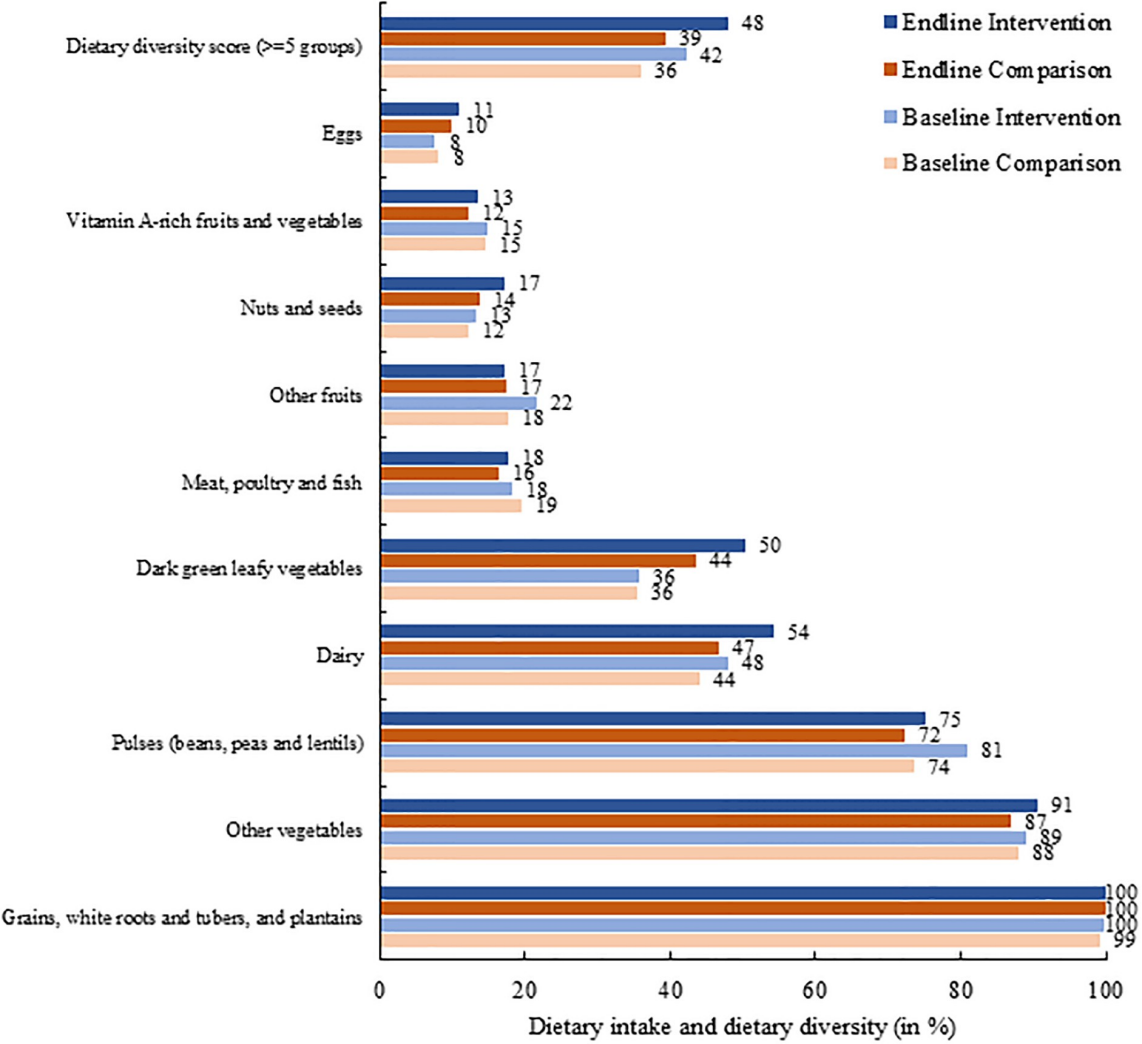
Dietary intake from various food groups (based on 24-hour recall) and practice of minimum dietary diversity (%) in comparison and intervention groups.

The mean domain specific scores for the comparison and intervention group have increased between the baseline and endline period (Table 2; S1 Table in S1 File). The average score for gender equitable roles domain (maximum score 72) has increased from 42.8 in baseline to 45.4 in endline for the comparison group and from 40.0 to 49.1 for the intervention group. The average score for adolescent diet and nutrition domain (maximum score 10) are on the lower side for both comparison (baseline, 3.62 and endline, 3.20) and intervention group (baseline, 2.97 and endline, 3.96) even as the latter show significant improvements. The dietary diversity score (i.e., intake from five or more food groups in the last 24 hours) among adolescents increased from 42% to 48% in the intervention group with greater effect on consumption of green leafy vegetables and nuts / seeds (Fig 1). It is worth noting that the potential dietary diversity based on the perception regarding what should be consumed by adolescents is considerably higher than the actual dietary diversity (S1 Table in S1 File).

**Table 2:** Mean of domain scores and Cohen’s *d* statistic for program effect in intervention group.

Domains	Baseline (Mean)		Endline (Mean)		Cohen’s *d*
	Comparison	Intervention	Comparison	Intervention	
Gender equitable roles	42.75	40.01	45.44	49.10	0.72
*Maximum score*: *72*	*(11*.*93)*	*(12*.*20)*	*(12*.*96)*	*(13*.*22)*	*[0*.*62; 0*.*82]*
Diet and nutrition	3.62	2.97	3.20	3.96	0.53
*Maximum score*: *10*	*(1*.*80)*	*(1*.*93)*	*(1*.*90)*	*(1*.*81)*	*[0*.*43; 0*.*62]*
Self-esteem	26.06	25.73	27.49	27.63	0.45
*Maximum score*: *32*	*(4*.*32)*	*(4*.*42)*	*(4*.*02)*	*(3*.*92)*	*[0*.*35; 0*.*55]*
Self-efficacy	27.64	27.45	28.91	29.25	0.38
*Maximum score*: *36*	*(4*.*59)*	*(4*.*96)*	*(4*.*44)*	*(4*.*41)*	*[0*.*28; 0*.*47]*
Employee rights and responsibilities	2.50	2.10	2.41	2.99	0.45
*Maximum score*: *13*	*(1*.*95)*	*(1*.*91)*	*(1*.*87)*	*(2*.*11)*	*[0*.*35; 0*.*54]*
N	1122	1205	1037	618	1823

Note: ***, ** and * denotes p-value significance at 1%, 5% and 10%, respectively.

Standard deviation of the mean scores is reported in parenthesis *(*.*)*.

The 95% confidence interval values are reported for Cohen’s *d* statistic in parenthesis *[*.*]*.

Further, the average knowledge score regarding employee rights and responsibilities (maximum score 13) is low but show increments for the adolescents in the intervention group (baseline, 2.1 and endline, 3.0). Small increments in scores are apparent in self-esteem and self-efficacy scores. The average score for the former increased from 25.7 in baseline to 27.6 during endline whereas for the latter it increased from 27.5 in baseline to 29.3 in endline.

Table 2 further reports the Cohen’s *d* value to provide insights on the effect size. This statistic describes the magnitude of the program impact for the intervention group and—as a general rule of thumb–effect size greater than 0.2 is considered to be of policy interest. The higher values can be further categorized to be of small (0.2 to 0.5), medium (0.5 to 0.8) or large (0.8 and above) impact. The program impact based on Cohen’s *d* is inferred to be of medium size for two domains of gender equitable roles (Cohen’s *d* 0.72 and 95% CI: 0.62; 0.82) and diet and nutrition (Cohen’s *d* 0.53 and 95% CI: 0.43; 0.62) and small size for three domains of self-esteem (Cohen’s *d* 0.45 and 95% CI: 0.35; 0.55), self-efficacy (Cohen’s *d* 0.38 and 95% CI: 0.28; 0.47) and employee rights / responsibilities (Cohen’s *d* 0.45 and 95% CI: 0.35; 0.54).

Logistic regression analysis is applied to examine the likelihood of better performance among the program participants (Table 3). PCA based domain specific quintiles for baseline and endline sample are constructed whereby those in top two quintiles are designated as better performers. The odds ratio (OR) reveal that program participants are more likely to be in the top two quintiles of domain scores. With baseline comparison group as the reference category, the program participants are 2.07 times (OR 2.07 and 95% CI: 1.69; 2.54) more likely to be in top two quintiles of the domain score in gender equitable roles. The odds ratio for other domains of diet and nutrition, self-efficacy, self-esteem and employee rights / responsibilities are 1.33 (95% CI: 1.08; 1.63), 1.74 (95% CI: 1.40; 2.16), 3.61 (95% CI: 2.84; 4.59) and 1.32 (95% CI: 1.07; 1.63), respectively.

**Table 3:** Logistic regression (odds ratio) and Poisson regression (incidence rate ratio) for higher domain scores (top two quintiles) for comparison and intervention groups.

Variables*	Gender equitable roles	Diet and nutrition	Self-esteem	Self-efficacy	Employee rights and responsibilities
Logistic regression	OR	OR	OR	OR	OR
Comparison (baseline) ^®^	1.00	1.00	1.00	1.00	1.00
95% CI	-	-	-	-	-
Intervention (baseline)	0.71***	0.57***	0.93	1.03	0.70***
95% CI	[0.60; 0.85]	[0.47; 0.68]	[0.74; 1.18]	[0.85; 1.24]	[0.58; 0.84]
Comparison (endline)	1.28***	0.66***	3.34***	1.59***	0.83*
95% CI	[1.07; 1.53]	[0.55; 0.80]	[2.69; 4.14]	[1.32; 1.92]	[0.69; 1.01]
Intervention (endline)	2.07***	1.33***	3.61***	1.74***	1.32**
95% CI	[1.69; 2.54]	[1.08; 1.63]	[2.84; 4.59]	[1.40; 2.16]	[1.07; 1.63]
Poisson regression	IRR	IRR	IRR	IRR	IRR
Comparison (baseline) ^®^	1.00	1.00	1.00	1.00	1.00
95% CI	-	-	-	-	-
Intervention (baseline)	0.94***	0.83***	0.99	1.00	0.84***
95% CI	[0.93; 0.95]	[0.79; 0.87]	[0.97; 1.00]	[0.98; 1.01]	[0.80; 0.89]
Comparison (endline)	1.06***	0.87***	1.06***	1.05***	0.94**
95% CI	[1.05; 1.08]	[0.83; 0.92]	[1.04; 1.08]	[1.03; 1.06]	[0.89; 1.00]
Intervention (endline)	1.15***	1.08***	1.06***	1.06***	1.16***
95% CI	[1.13; 1.17]	[1.02; 1.14]	[1.04; 1.08]	[1.04; 1.08]	[1.09; 1.23]

Note:

***, ** and * denotes p-value significance at 1%, 5% and 10%, respectively.

The models are adjusted for age of the adolescent, schooling status, maternal and paternal education, household size, household occupation, family type, social group, poverty status, household construction material and household assets-based wealth quintile.

The bottom panel of Table 3 reports the incidence rate ratio (IRR) from the Poisson regression model and confirms a positive and significant impact of program participation on domains scores. Compared to the reference category, the expected gender equitable roles scale score of program participants is enhanced by 15% (i.e., a factor of 1.15 with 95% CI: 1.13; 1.17). Similarly, the domain scores of the participants in diet and nutrition, self-efficacy, self-esteem and employee rights / responsibilities is increased by 8% (IRR 1.08: 95% CI: 1.13; 1.17), 6% (IRR 1.06: 95% CI: 1.04; 1.08), 6% (IRR 1.06: 95% CI: 1.04; 1.08) and 16% (IRR 1.16: 95% CI: 1.09; 1.23), respectively. The unadjusted logistic and Poisson regression models also provide same inferences (S3 Table in S1 File).

The results for the socioeconomic correlates from the adjusted logistic and Poisson regression are reported in S4, S5 Tables in S1 File, respectively. Age of the adolescent girl has a positive association with domain scores of gender equitable roles, diet and nutrition and employee rights / responsibilities. Those who are currently in school are also likely to have higher domain scores for diet and nutrition (OR 1.75; IRR 1.20; p-value < 0.00), employee rights / responsibilities (OR 1.58; IRR 1.24; p-value < 0.00) and self-efficacy (OR 1.79; IRR 1.07; p-value < 0.00). Maternal and paternal education are also associated with domain scores with the former displaying a stronger association. Social group or religious affiliation have no particular effect on domain scores. Adolescent girls from economically better-off households have relatively higher IRR of better scores and the effect is more prominent in case of diet and nutrition (IRR 1.10; p-value < 0.00).

The DID analysis confirms a significant impact of the program on the exposed participants (Table 4). The unadjusted effects are statistically significant for all the domains whereas the adjusted DID estimates for program participation are statistically significant for three domains viz. gender equitable roles (coefficient 6.30; p-value < 0.00), diet and nutrition (coefficient 1.37; p-value < 0.00) and employee rights / responsibilities score (coefficient 0.95; p-value < 0.00). The DID estimates on self-esteem and self-efficacy were positive but lacked statistical significance. Nevertheless, the average scores for self-esteem and self-efficacy for the groups are relatively higher and closer to the maximum domain scores of 32 and 36, respectively.

**Table 4:** Impact estimates based on difference-in-differences and propensity score matching analysis.

Domains	DID Coefficient		PSM Coefficient	
	(Unadjusted)	(Adjusted)	ATE	ATT
Gender equitable roles	6.40***	6.30***	3.74***	3.32***
	*(0*.*83)*	*(0*.*84)*	*(0*.*72)*	*(0*.*88)*
Diet and nutrition	1.41***	1.37***	0.80***	0.72***
	*(0*.*12)*	*(0*.*12)*	*(0*.*11)*	*(0*.*12)*
Self-esteem	0.47*	0.41	-0.02	0.05
	*(0*.*27)*	*(0*.*27)*	*(0*.*23)*	*(0*.*26)*
Self-efficacy	0.53*	0.41	0.51**	0.28
	*(0*.*30)*	*(0*.*29)*	*(0*.*26)*	*(0*.*28)*
Employee rights and responsibilities	0.98***	0.95***	0.69***	0.59***
	*(0*.*13)*	*(0*.*13)*	*(0*.*13)*	*(0*.*14)*
N	3982	3982	1655	1655

Note:

***, ** and * denotes p-value significance at 1%, 5% and 10%, respectively.

Standard error of the DID coefficient and the PSM estimates is reported in parenthesis *(*.*)*. The PSM based ATE and ATT coefficients are based on analysis of comparison and intervention group domain scores at endline.

Table 4 also reports the PSM based ATE and ATT coefficients to discern the impact of the program at endline vis-à-vis the baseline period. The model satisfies the covariate balancing tests and checks for common support for the propensity scores. The propensity scores mean and standard deviation is estimated to be 0.304 and 0.066, respectively. The common support region for the propensity scores ranges from 0.128 to 0.505. Similar to the DID inference, the ATE is found to be significant for all the domains except self-esteem whereas the ATT based inference of program impact was similar to that derived from the adjusted DID models. The ATT coefficient reveal a positive change of 3.32 (p-value < 0.00) in the domain score of gender equitable roles, 0.72 (p-value < 0.00) for diet and nutrition score and 0.59 (p-value < 0.00) for employee rights / responsibilities score. The ATE and ATT estimates based on the endline sample of intervention and comparison group also yield similar conclusions (S6 Table in S1 File).

## 5. Discussion

This study evaluates the impact of delivering PACE++ curriculum for adolescent girls in community settings of rural Bihar. Based on the quasi-experimental study design, the study focused on domains related to gender attitudes, empowerment and dietary practices. The five salient findings of the study are as follows. First, TARA intervention had a positive impact on domain scores of intervention group comprising of adolescent girls who participated in all or most of program meetings and events. Second, the DID and PSM analyses confirm that the impact are specifically significant for the domains of attitudes toward gender equity norms, nutritional knowledge and understanding of employee related rights and responsibilities. Third, the effect size–as discerned through Cohen’s d–is of policy interest as all the domain scores show small to medium size program impact on the intervention group. Fourth, the intervention group demonstrated a positive change in dietary diversity practices whereby consumption from five or more food groups increased significantly over the intervention period. Fourth, maternal education and household wealth status have an independent influence on domain scores. In particular, maternal education is found to have significant association with empowerment related scales of self-esteem and self-efficacy. Household wealth status has a positive bearing on expected score of dietary practices. Finally, the expected domain scores of school-going adolescent girls is higher than those who have discontinued formal education.

The study, however, is not devoid of limitations. First, during endline about two-third of the intervention area sample reported greater exposure to the program and was treated as the intervention group. This exposure-based attrition of sample may bias significance of program impact across domain scores. The adopted sampling approach, however, was necessary to check for overall program participation and to ensure that the sample was drawn randomly from the community and was not necessarily restricted to the program participants to discern broader implications of the program. Nevertheless, the analysis focused only on regular participants to estimate the magnitude of the impact. Second, the construction of the domain scores relied on established scales for the domains related to gender equity (GEM scale) and empowerment (self-esteem and self-efficacy scale). The scale for diet and nutrition apply well-established dietary diversity indicators and knowledge of most widely prevalence micronutrient deficiency (anemia). Whereas in the absence of relevant scale for employment related indicators, the items reflecting employee rights and responsibilities was utilized that provide a balance between awareness of employee duties and being informed regarding basic rights to safeguard employees from exploitation. This also suggests a need for developing valid and reliable scale for adolescents to assess the effect of like-skills training on employability in developing country contexts [33]. Third, it is important to acknowledge that TARA intervention was disrupted because of COVID-19 pandemic and the subsequent restrictions imposed on community-based engagements. This is likely to have curtailed the full potential of the trainings and might also have deterred some from active participation in group events. Furthermore, the study is based on a pre- and post-design. One of the limitations of this approach is in establishing causality. It may be difficult to attribute the changes solely to the intervention as other cofounding variables can influence the results.

Life skills–particularly soft skills related to self-efficacy, communication and leadership—are inextricably linked to equity and empowerment whose relevance increases manifold for women and girls across the developing world [34, 35]. However, a vast majority of women and girls in resource-poor rural settings are located outside the purview of the formal sector and their agency is barely recognized in social lives and their work participation as an economic contribution for the household or the community [36–38]. Informality in economic set up further restricts the avenues for engagement and empowerment of women and girls [39]. Lack of gender mainstreaming and gender-neutral social platforms also complicates the environment to impart trainings for development of life skills among women and girls [40]. The TARA intervention, therefore, assumes more relevance as efforts to deliver such life-skills trainings can lead to greater empowerment during adolescence [19].

The TARA intervention aimed at leveraging the PACE curriculum and delivering it in a rural community setting that has wide policy relevance in the Indian context. Given the large scale of the informal sector, there might be limited work platforms to provide such training modules for adolescent girls. The SHG platforms that are supported by the government provide an important avenue to empower adolescent girls, develop their resilience and support their aims and aspirations [41]. In particular, there is further scope to improve areas related to diet and nutrition knowledge, awareness and practices [42]. As such, there is a dearth of evidence on dietary practices of adolescent girls in India and it is well-known that the nutritional status has huge bearing on reproductive as well as maternal and child health [43, 44]. Besides, linking adolescent girls to JEEViKA forums is a reasonable strategy for future development as it has considerable impact on women empowerment and their skills and capabilities [45].

The program impact size of the intervention is of policy interest as it demonstrates possibility of improving adolescent well-being through outreach based on community platforms. The effect size as discerned by Cohen’s d is encouraging enough to be considered for wider scale up of such initiatives using existing platforms such as JEEViKA and other self-help group and adolescent forums. The effect size is also comparable to similar to other adolescent girl programmes delivered through school-based initiatives [19]. The boost in life-skills is a central argument of such programs as it is a fundamental determinant of a range of soft skills such as communication and coordination that are highly relevant for successful career as well as entrepreneurial abilities and economic leadership [7, 46]. Nevertheless, these programs have to be contextually relevant and well-implemented else there are risk of underestimation of its effects along with possible risks of witnessing a null effect [47].

The program impact of TARA intervention is driven by a sound theory of change rooted in the positive youth development framework [13, 14, 15]. The intervention thus emphasized on the need for action on three fundamental areas of concern viz. a) restrictive social norms for girls and women, b) poor individual level engagement and learning and c) lack of appropriate platforms. Strategies were put in place to empower adolescent girls through expression of agency and aspirations, improving scope for work and entrepreneurship and by enhancing awareness on health and nutrition. These activities were expected to be strengthened with broader community support from peers, especially the members of the SAC and JEEViKA groups as well as improved departmental linkages for accessing government schemes and entitlements. With active mobilization efforts and capacity building initiatives it is expected that a range of skills will be developed which will ultimately influence the ability of adolescent girls to achieve their personal goals and simultaneously help improve the status of women empowerment and gender equity in the community [48, 49].

It is worth noting that the impact of the intervention on domain scores pertaining to self-esteem and self-efficacy is relatively small. This finding is partly associated with higher baseline scores on these indicators whereas greater improvements in self-esteem are noted especially when the initial scores are lower [50]. Such pattern is not unusual as studies find that self-esteem usually remains consistent across the different phases of adolescence and are partly associated with other personality traits [51]. Besides, self-efficacy and self-esteem are usually correlated with the former being more effective in influencing performance and accomplishments longitudinally [52–54]. Here also the findings show a significant correlation between these scores in both groups and across baseline and endline period. Continued focus on self-efficacy, therefore, is an important area for further strengthening of such interventions improvements as it holds wider implications on resourcefulness and psychological well-being during adolescence [55].

Before concluding, it is also important to outline the lessons learnt from program intervention during the pandemic period. The direct engagement with TARA groups immediately alerted the wide sense of fear and anxiety among adolescents that manifested in psychosocial and mental health concerns. The impact could be disproportionately on adolescent girls from economically unprivileged background. The COVID-19 protocols also implied that all efforts were counselling and information sharing were to be through digital mediums and its efficacy can be a challenge for those lacking access to mobile devices and internet. This also curtailed the program effect as the results demonstrate greater benefits for those who were regular participants. Such impact, however, was unavoidable and similar concerns are widespread across other remote and rural settings of India that have weak IT infrastructure as well as poor means for digital communication [56]. This also offers lessons for considering stress management as an important component of life skills training. Although, the TARA intervention introduced stress management sessions in the post-COVID phase but it is important that this can be considered as a regular part of the PACE++ curriculum or under similar other programs in future. The COVID-19 disruption also meant limited scope and engagements for boosting social capital of TARA clubs by strengthening linkages with local social action committees (SACs) for access to relevant and updated information on government schemes and entitlements for girls. The program exposure, however, has opened up avenues for TARA clubs to connect with community-based organizations and benefit from convergence-based training and livelihood development programmes of the government.

## 6. Conclusion

The TARA intervention contributed to the development of the strategies for engaging and empowering adolescent girls through augmenting health and nutrition issues in the widely used PACE curriculum and also implementing this in community-based settings. Both the changes are extremely important as adolescent well-being cannot be discussed in isolation with health and nutrition concerns and also that reaching out to these in rural settings warrants a community centric platform as school settings may have both time and participation constraints. It is encouraging that the intervention shows a discernible impact of PACE++ curriculum training on a range of indicators associated with women empowerment and adolescent health and nutrition. The intervention also highlights the feasibility of delivering the modified PACE curriculum in rural settings and the scope for leveraging community platforms such as SHGs for program roll out. This provides further opportunities to scale up these through government initiatives such as SRLM and JEEViKA. The findings overall are encouraging but they also reveal new challenges such as dietary practices among adolescent girls that has huge implications on population health and well-being. Notwithstanding program impact and effect size, greater caution is warranted while scaling up of such models. In particular, appropriate contextualization of the session tools and implementation techniques has an instrumental role to play. Before concluding, it is worth reiterating the adverse effects that adversities such as pandemics can have on adolescent lives. During such testing times the social capital of adolescent group forums and platforms can be of high relevance and support. To conclude, the TARA intervention showcases the importance of delivering the modified PACE++ curriculum in rural settings through leveraging community platforms. The linkages of the program with established government institutions and initiatives boost the sustainability prospects of the initiative. The pilot, therefore, provides robust evidence for upscaling, replicating and promoting similar programs to reach out to all adolescent girls in India and elsewhere.

## Supporting information

S1 Checklist (DOC)

S1 Data (ZIP)

S1 File (DOCX)
