# Supplementary material for: Delivering PACE++ curriculum in community settings: Impact of TARA intervention on gender attitudes and dietary practices among adolescent girls in Bihar, India
Source: PLoS One. 2023 Nov 3;18(11):e0293941. doi: 10.1371/journal.pone.0293941 (PMC10624310; doi:10.1371/journal.pone.0293941)
Supplement: S1 File — (DOCX) [file pone.0293941.s003.docx]

**Supplementary Table S1**: Mean score of domain items for control and treatment groups, baseline and endline period

|  | **Domain and Items** | **Baseline** | | **Endline** | |
| --- | --- | --- | --- | --- | --- |
|  |  | Comparison | Intervention | Comparison | Intervention |
|  | ***Diet and Nutrition Score*** | 3.62 | 2.97 | 3.21 | 3.96 |
| i) | Intake of adequate dietary diverse food | 0.36 | 0.42 | 0.39 | 0.48 |
| ii) | Knowledge of adequate dietary diversity | 0.82 | 0.71 | 0.77 | 0.84 |
| iii) | Heard about iron-deficiency anemia | 0.79 | 0.65 | 0.69 | 0.82 |
| iv) | Anemia symptoms knowledge - Low energy / Weakness | 0.52 | 0.39 | 0.43 | 0.56 |
| v) | Anemia symptoms knowledge - Paleness / Pallor | 0.36 | 0.24 | 0.29 | 0.40 |
| vi) | Anemia symptoms knowledge - Spoon / Bent nails | 0.00 | 0.01 | 0.01 | 0.03 |
| vii) | Anemia symptoms knowledge - More likely to be sick | 0.07 | 0.05 | 0.06 | 0.08 |
| viii) | Causes of anemia - Lack of iron in diet | 0.57 | 0.41 | 0.45 | 0.61 |
| ix) | Causes of anemia - sickness / infection (malaria etc.) | 0.09 | 0.06 | 0.08 | 0.08 |
| x) | Causes of anemia - Heavy menstrual bleeding | 0.05 | 0.03 | 0.02 | 0.06 |
|  | ***Self-Esteem*** | 26.06 | 25.73 | 27.48 | 27.63 |
| i) | On the whole, I am satisfied with myself | 3.66 | 3.53 | 3.68 | 3.74 |
| ii) | At times I think I am not good at all (reverse scoring) | 1.80 | 1.91 | 2.88 | 2.86 |
| iii) | I feel that I have a number of good qualities | 3.17 | 3.10 | 3.26 | 3.19 |
| iv) | I am able to do things as well as other people | 3.69 | 3.63 | 3.68 | 3.66 |
| v) | I feel I have things to be proud of | 3.20 | 3.14 | 3.33 | 3.40 |
| vi) | I respect myself | 3.79 | 3.70 | 3.81 | 3.78 |
| vii) | I believe I can be a strong leader | 3.42 | 3.41 | 3.52 | 3.58 |
| viii) | I think others value me | 3.31 | 3.29 | 3.33 | 3.43 |
|  | ***Self-Efficacy*** | 27.64 | 27.45 | 28.90 | 29.25 |
| i) | I can choose how to spend my free time | 3.64 | 3.57 | 3.69 | 3.73 |
| ii) | I can go for higher studies if I wish to, despite of any opposition | 3.20 | 3.20 | 3.33 | 3.42 |
| iii) | I can choose to earn an income if I wish to | 3.57 | 3.45 | 3.68 | 3.58 |
| iv) | I can talk freely to my parents about my aspirations | 3.73 | 3.61 | 3.70 | 3.68 |
| v) | I can express choice in type of clothing for myself | 3.39 | 3.33 | 3.42 | 3.52 |
| vi) | I do not feel any shame around the time I have my menstruation | 1.92 | 2.11 | 2.44 | 2.57 |
| vii) | I can provide financial support to my parents in old age | 3.88 | 3.83 | 3.87 | 3.86 |
| viii) | I can go to the market, cinema, fair, or other place of entertainment with my friends | 2.36 | 2.36 | 2.57 | 2.61 |
| ix) | I can freely express choice in whom I want to marry | 1.94 | 1.99 | 2.20 | 2.29 |
|  | ***Employee Rights and Responsibilities*** | 2.50 | 2.10 | 2.40 | 2.99 |
| i) | Right to fair compensation | 0.40 | 0.30 | 0.37 | 0.41 |
| ii) | Right to privacy | 0.02 | 0.02 | 0.02 | 0.02 |
| iii) | Right to freedom from discrimination | 0.08 | 0.06 | 0.08 | 0.15 |
| iv) | Right to safe workplace | 0.02 | 0.02 | 0.02 | 0.04 |
| v) | Right to harassment free workplace | 0.01 | 0.01 | 0.02 | 0.03 |
| vi) | Participation in team work | 0.08 | 0.05 | 0.07 | 0.08 |
| vii) | Following workplace discipline | 0.40 | 0.33 | 0.35 | 0.47 |
| viii) | Maintaining quality of work | 0.28 | 0.25 | 0.30 | 0.37 |
| ix) | Taking initiative and responsibility | 0.31 | 0.29 | 0.31 | 0.34 |
| x) | Honesty as an employee | 0.36 | 0.37 | 0.37 | 0.47 |
| xi) | Loyalty as an employee | 0.04 | 0.05 | 0.05 | 0.06 |
| xii) | Accountability as an employee | 0.04 | 0.04 | 0.05 | 0.07 |
| xiii) | Knowledge of any employee rights | 0.45 | 0.32 | 0.41 | 0.46 |
|  | ***Gender equitable roles*** | 42.75 | 40.01 | 45.45 | 49.10 |
| i) | Men need more care as they work harder than women. | 2.86 | 2.59 | 1.98 | 2.42 |
| ii) | It is necessary to give dowry gender discrimination | 1.91 | 1.76 | 3.15 | 3.21 |
| iii) | Boys are naturally better at math and science than girls | 1.58 | 1.56 | 3.14 | 3.33 |
| iv) | Boys are naturally better than girls in sports. gender discrimination | 1.78 | 1.69 | 3.01 | 3.18 |
| v) | It is a girl’s fault if a male student or teacher sexually harasses her | 1.30 | 1.27 | 3.46 | 3.50 |
| vi) | Giving children a bath and feeding children are the mother’s responsibility. | 3.40 | 3.10 | 1.76 | 2.11 |
| vii) | There are times a woman deserves to be beaten | 1.48 | 1.40 | 3.28 | 3.27 |
| viii) | It is alright for a man to beat his wife | 1.17 | 1.17 | 3.74 | 3.78 |
| ix) | A woman should tolerate violence in order to keep her family together | 2.73 | 2.53 | 2.26 | 2.57 |
| x) | A man using violence against his wife is a private matter that should not be discussed outside the couple | 2.83 | 2.55 | 2.23 | 2.42 |
| xi) | A man should have the final word on decisions in his home | 2.54 | 2.28 | 2.46 | 2.75 |
| xii) | The husband should decide what major household items to buy | 2.34 | 2.23 | 2.58 | 2.95 |
| xiii) | A woman should obey her husband in all things | 3.07 | 2.97 | 1.74 | 2.02 |
| xiv) | Man should get the best share of the food in the family | 2.94 | 2.70 | 2.08 | 2.39 |
| xv) | Man should have the final say in what should be cooked today | 2.37 | 2.20 | 2.48 | 2.84 |
| xvi) | Man do not need to inform his wife regarding his days or personal expenses | 1.54 | 1.66 | 2.93 | 2.92 |
| xvii) | Woman should always inform her husband about the financial expenses she has made | 3.52 | 3.24 | 1.57 | 1.61 |
| xviii) | Woman should always seek husband’s permission for any expenses | 3.39 | 3.11 | 1.59 | 1.84 |

Note: Self-esteem categories consisted of five scores ranging from strongly disagree to strongly agree. The respondents reporting agree or strongly agree are considered to be reporting positively.

**Supplementary Table S2**: Cronbach’s alpha coefficient of domain scales for comparison and treatment groups

| Domains | Baseline | | | Endline | | | Overall |
| --- | --- | --- | --- | --- | --- | --- | --- |
|  | Comparison | Treatment | All | Comparison | Treatment | All |  |
| Diet and nutrition | 0.61 | 0.67 | 0.65 | 0.65 | 0.60 | 0.65 | 0.65 |
| Self-esteem | 0.59 | 0.64 | 0.62 | 0.65 | 0.67 | 0.64 | 0.63 |
| Self-efficacy | 0.58 | 0.65 | 0.62 | 0.60 | 0.62 | 0.60 | 0.62 |
| Employee rights and responsibilities | 0.61 | 0.64 | 0.63 | 0.57 | 0.63 | 0.61 | 0.62 |
| Gender equitable roles | 0.84 | 0.84 | 0.84 | 0.86 | 0.87 | 0.87 | 0.78 |

**Supplementary Table S3**: Unadjusted logistic regression (odds ratio) and Poisson regression (incidence rate ratio) for higher domain scores (top two quintiles) for comparison and intervention groups

| Variables* | Gender equitable roles | Diet and nutrition | Self-esteem | Self-efficacy | Employee rights and responsibilities |
| --- | --- | --- | --- | --- | --- |
| Logistic regression | OR | OR | OR | OR | OR |
| Comparison (baseline) ® | 1.00 | 1.00 | 1.00 | 1.00 | 1.00 |
| 95% CI | - | - | - | - | - |
| Intervention (baseline) | 0.71*** | 0.57*** | 0.95 | 1.00 | 0.71*** |
| 95% CI | [0.60; 0.85] | [0.47; 0.67] | [0.75; 1.20] | [0.83; 1.19] | [0.58; 0.84] |
| Comparison (endline) | 1.28*** | 0.69*** | 3.27*** | 1.59*** | 0.90 |
| 95% CI | [1.07; 1.52] | [0.58; 0.83] | [2.65; 4.03] | [1.33; 1.90] | [0.75; 1.08] |
| Intervention (endline) | 2.08*** | 1.34*** | 3.59*** | 1.72*** | 1.44*** |
| 95% CI | [1.70; 2.54] | [1.09; 1.63] | [2.84; 4.53] | [1.40; 2.11] | [1.18; 1.77] |
|  |  |  |  |  |  |
| Poisson regression | IRR | IRR | IRR | IRR | IRR |
| Comparison (baseline) ® | 1.00 | 1.00 | 1.00 | 1.00 | 1.00 |
| 95% CI | - | - | - | - | - |
| Intervention (baseline) | 0.94*** | 0.82*** | 0.99 | 1.00 | 0.84*** |
| 95% CI | [0.92; 0.95] | [0.78; 0.86] | [0.97; 1.00] | [0.98; 1.01] | [0.80; 0.89] |
| Comparison (endline) | 1.06*** | 0.89*** | 1.05*** | 1.05*** | 0.96 |
| 95% CI | [1.05; 1.08] | [0.85; 0.93] | [1.04; 1.08] | [1.03; 1.06] | [0.89; 1.00] |
| Intervention (endline) | 1.15*** | 1.09*** | 1.06*** | 1.06*** | 1.19*** |
| 95% CI | [1.13; 1.17] | [1.04; 1.15] | [1.04; 1.08] | [1.04; 1.08] | [1.13; 1.27] |

Note: ***, ** and * denotes p-value significance at 1%, 5% and 10%, respectively. N = 3985.

The models are adjusted for age of the adolescent, schooling status, maternal and paternal education, household size, household occupation, family type, social group, poverty status, household construction material and household assets-based wealth quintile.

**Supplementary Table S4**: Logistic regression based adjusted odds ratio for higher domain scores (top two quintiles) for comparison and treatment groups

| Variables | Diet and nutrition | | Gender equitable roles | | Employee rights and responsibilities | | Self-efficacy | | Self-esteem | |
| --- | --- | --- | --- | --- | --- | --- | --- | --- | --- | --- |
|  | OR | SE | OR | SE | OR | SE | OR | SE | OR | SE |
| Comparison (baseline) ® | 1.00 |  | 1.00 |  | 1.00 |  | 1.00 |  | 1.00 |  |
| Treatment (baseline) | 0.57*** | 0.05 | 0.71*** | 0.07 | 0.70*** | 0.07 | 1.03 | 0.10 | 0.93 | 0.11 |
| Comparison (endline) | 0.66*** | 0.06 | 1.28*** | 0.12 | 0.83* | 0.08 | 1.59*** | 0.15 | 3.34*** | 0.37 |
| Treatment (endline) | 1.33*** | 0.14 | 2.07*** | 0.22 | 1.32** | 0.14 | 1.74*** | 0.19 | 3.61*** | 0.44 |
| Age (15 years) ® | 1.00 |  | 1.00 |  | 1.00 |  | 1.00 |  | 1.00 |  |
| 16 years | 1.22** | 0.12 | 0.98 | 0.10 | 1.29** | 0.13 | 1.18 | 0.12 | 0.99 | 0.11 |
| 17 years | 1.23** | 0.13 | 1.08 | 0.11 | 1.34*** | 0.14 | 1.03 | 0.11 | 1.01 | 0.12 |
| 18 years | 1.12 | 0.13 | 1.14 | 0.13 | 1.34** | 0.16 | 1.14 | 0.13 | 0.95 | 0.12 |
| 19 years | 1.43*** | 0.17 | 1.00 | 0.12 | 1.56*** | 0.19 | 1.32** | 0.16 | 0.98 | 0.13 |
| Attending school (No) ® | 1.00 |  | 1.00 |  | 1.00 |  | 1.00 |  | 1.00 |  |
| Attending school (Yes) | 1.75*** | 0.20 | 1.14 | 0.12 | 1.58*** | 0.18 | 1.79*** | 0.21 | 1.12 | 0.13 |
| Mother – Illiterate ® | 1.00 |  | 1.00 |  | 1.00 |  | 1.00 |  | 1.00 |  |
| Mother – Primary level | 1.19** | 0.10 | 0.89 | 0.07 | 1.36*** | 0.12 | 1.23** | 0.11 | 1.13 | 0.11 |
| Mother – Above primary | 1.63*** | 0.16 | 1.05 | 0.10 | 1.53*** | 0.15 | 1.74*** | 0.17 | 1.23* | 0.14 |
| Father – Illiterate ® | 1.00 |  | 1.00 |  | 1.00 |  | 1.00 |  | 1.00 |  |
| Father – Primary level | 1.23* | 0.13 | 0.91 | 0.09 | 1.13 | 0.12 | 1.24** | 0.13 | 1.12 | 0.13 |
| Father – Above primary | 1.26** | 0.12 | 0.95 | 0.09 | 1.26** | 0.12 | 1.22** | 0.12 | 1.01 | 0.11 |
| Household size (=< 4) ® | 1.00 |  | 1.00 |  | 1.00 |  | 1.00 |  | 1.00 |  |
| Household size > 4 | 0.98 | 0.10 | 1.13 | 0.11 | 1.07 | 0.11 | 0.94 | 0.10 | 1.03 | 0.12 |
| Social group – SC/ST ® | 1.00 |  | 1.00 |  | 1.00 |  | 1.00 |  | 1.00 |  |
| OBC | 1.15 | 0.10 | 1.05 | 0.09 | 1.10 | 0.10 | 1.07 | 0.10 | 0.83* | 0.08 |
| Others | 1.54** | 0.28 | 1.03 | 0.19 | 1.26 | 0.23 | 1.14 | 0.21 | 0.91 | 0.19 |
| Religion (Hinduism) ® | 1.00 |  | 1.00 |  | 1.00 |  | 1.00 |  | 1.00 |  |
| Muslim | 1.26 | 0.22 | 0.91 | 0.16 | 1.00 | 0.18 | 1.01 | 0.18 | 1.32 | 0.25 |
| Others | 0.35 | 0.38 | 0.9 | 0.67 | 0.40 | 0.43 | 0.68 | 0.57 | 0.76 | 0.64 |
| Occupation (self-employed) ® | 1.00 |  | 1.00 |  | 1.00 |  | 1.00 |  | 1.00 |  |
| Regular salaried | 1.04 | 0.09 | 0.89 | 0.08 | 1.19* | 0.11 | 0.99 | 0.09 | 1.11 | 0.11 |
| Casual labor | 0.71*** | 0.07 | 1.02 | 0.09 | 0.92 | 0.09 | 0.87 | 0.08 | 0.97 | 0.10 |
| Unemployed | 0.21** | 0.14 | 1.00 | 0.49 | 1.37 | 0.68 | 0.51 | 0.26 | 1.04 | 0.54 |
| Family type (Nuclear) ® | 1.00 |  | 1.00 |  | 1.00 |  | 1.00 |  | 1.00 |  |
| Joint family | 1.14* | 0.09 | 0.99 | 0.07 | 0.90 | 0.07 | 1.00 | 0.08 | 1.03 | 0.09 |
| Above poverty line ® | 1.00 |  | 1.00 |  | 1.00 |  | 1.00 |  | 1.00 |  |
| Below poverty line | 1.20** | 0.09 | 1.13* | 0.08 | 1.25*** | 0.10 | 1.07 | 0.08 | 1.05 | 0.09 |
| House (non-durable material) ® | 1.00 |  | 1.00 |  | 1.00 |  | 1.00 |  | 1.00 |  |
| Durable housing | 1.07 | 0.09 | 0.92 | 0.07 | 1.15 | 0.10 | 1.18** | 0.10 | 0.90 | 0.08 |
| Wealth quintile (Lowest) ® | 1.00 |  | 1.00 |  | 1.00 |  | 1.00 |  | 1.00 |  |
| Second | 1.02 | 0.12 | 1.08 | 0.12 | 0.97 | 0.11 | 1.05 | 0.13 | 1.06 | 0.14 |
| Middle | 1.00 | 0.12 | 0.90 | 0.10 | 1.00 | 0.12 | 1.23* | 0.15 | 1.24 | 0.16 |
| Fourth | 1.12 | 0.14 | 0.92 | 0.11 | 0.89 | 0.12 | 1.46*** | 0.19 | 0.91 | 0.13 |
| Highest | 1.15 | 0.16 | 1.15 | 0.15 | 1.02 | 0.14 | 1.67*** | 0.23 | 1.10 | 0.17 |

Note: *** p<0.01, ** p<0.05, * p<0.1

**Supplementary Table S5**: Poisson regression-based incidence rate ratio for domain scores of comparisons and treatment groups

| Variables | Diet and nutrition | | Gender equitable roles | | Employee rights and responsibilities | | Self-efficacy | | Self-esteem | |
| --- | --- | --- | --- | --- | --- | --- | --- | --- | --- | --- |
|  | IRR | SE | IRR | SE | IRR | SE | IRR | SE | IRR | SE |
| Comparison (baseline) ® |  |  |  |  |  |  |  |  |  |  |
| Treatment (baseline) | 0.83*** | 0.02 | 0.94*** | 0.01 | 0.84*** | 0.02 | 1.00 | 0.01 | 0.99 | 0.01 |
| Comparison (endline) | 0.87*** | 0.02 | 1.06*** | 0.01 | 0.94** | 0.03 | 1.05*** | 0.01 | 1.06*** | 0.01 |
| Treatment (endline) | 1.08*** | 0.03 | 1.15*** | 0.01 | 1.16*** | 0.04 | 1.06*** | 0.01 | 1.06*** | 0.01 |
| Age (15 years) ® |  |  |  |  |  |  |  |  |  |  |
| 16 years | 1.08*** | 0.03 | 1.00 | 0.01 | 1.11*** | 0.03 | 1.01 | 0.01 | 1.00 | 0.01 |
| 17 years | 1.10*** | 0.03 | 1.00 | 0.01 | 1.13*** | 0.03 | 1.01 | 0.01 | 1.00 | 0.01 |
| 18 years | 1.09*** | 0.03 | 1.02** | 0.01 | 1.10*** | 0.04 | 1.01 | 0.01 | 1.00 | 0.01 |
| 19 years | 1.11*** | 0.03 | 1.00 | 0.01 | 1.17*** | 0.04 | 1.02* | 0.01 | 1.00 | 0.01 |
| Attending school (No) ® |  |  |  |  |  |  |  |  |  |  |
| Attending school (Yes) | 1.20*** | 0.03 | 1.02** | 0.01 | 1.24*** | 0.04 | 1.07*** | 0.01 | 1.04*** | 0.01 |
| Mother – Illiterate ® |  |  |  |  |  |  |  |  |  |  |
| Mother – Primary level | 1.09*** | 0.02 | 0.98*** | 0.01 | 1.15*** | 0.03 | 1.02** | 0.01 | 1.01 | 0.01 |
| Mother – Above primary | 1.16*** | 0.03 | 1.00 | 0.01 | 1.22*** | 0.04 | 1.05*** | 0.01 | 1.02** | 0.01 |
| Father – Illiterate ® |  |  |  |  |  |  |  |  |  |  |
| Father – Primary level | 1.04 | 0.03 | 0.99 | 0.01 | 1.06* | 0.03 | 1.02* | 0.01 | 1.01 | 0.01 |
| Father – Above primary | 1.06** | 0.03 | 0.99** | 0.01 | 1.11*** | 0.03 | 1.02* | 0.01 | 1.00 | 0.01 |
| Household size (=< 4) ® |  |  |  |  |  |  |  |  |  |  |
| Household size > 4 | 1.00 | 0.03 | 1.02** | 0.01 | 1.04 | 0.03 | 1.00 | 0.01 | 1.01 | 0.01 |
| Social group – SC/ST ® |  |  |  |  |  |  |  |  |  |  |
| OBC | 1.07*** | 0.02 | 1.00 | 0.01 | 1.04 | 0.03 | 1.01 | 0.01 | 0.99 | 0.01 |
| Others | 1.09** | 0.05 | 1.00 | 0.01 | 1.09 | 0.06 | 0.99 | 0.02 | 0.99 | 0.02 |
| Religion (Hinduism) ® |  |  |  |  |  |  |  |  |  |  |
| Muslim | 1.04 | 0.05 | 1.04*** | 0.01 | 1.01 | 0.05 | 1.00 | 0.02 | 1.01 | 0.02 |
| Others | 0.85 | 0.19 | 1.06 | 0.05 | 0.79 | 0.21 | 0.98 | 0.07 | 0.96 | 0.07 |
| Occupation (self-employed) ® |  |  |  |  |  |  |  |  |  |  |
| Regular salaried | 1.04* | 0.02 | 0.97*** | 0.01 | 1.04 | 0.03 | 1.01 | 0.01 | 1.01 | 0.01 |
| Casual labor | 0.92*** | 0.02 | 0.99* | 0.01 | 0.92*** | 0.03 | 0.99 | 0.01 | 1.00 | 0.01 |
| Unemployed | 0.69** | 0.10 | 1.02 | 0.04 | 1.04 | 0.14 | 0.96 | 0.04 | 0.98 | 0.05 |
| Family type (Nuclear) ® |  |  |  |  |  |  |  |  |  |  |
| Joint family | 1.04** | 0.02 | 1.01 | 0.01 | 0.95** | 0.02 | 1.00 | 0.01 | 1.00 | 0.01 |
| Above poverty line ® |  |  |  |  |  |  |  |  |  |  |
| Below poverty line | 1.06*** | 0.02 | 1.02*** | 0.01 | 1.05** | 0.02 | 1.00 | 0.01 | 1.00 | 0.01 |
| House (non-durable material) ® |  |  |  |  |  |  |  |  |  |  |
| Durable housing | 1.02 | 0.02 | 0.98*** | 0.01 | 1.05* | 0.03 | 1.01 | 0.01 | 0.99* | 0.01 |
| Wealth quintile (Lowest) ® |  |  |  |  |  |  |  |  |  |  |
| Second | 1.02 | 0.03 | 1.02** | 0.01 | 1.03 | 0.04 | 1.00 | 0.01 | 1.01 | 0.01 |
| Middle | 1.03 | 0.03 | 1.00 | 0.01 | 1.04 | 0.04 | 1.02** | 0.01 | 1.04*** | 0.01 |
| Fourth | 1.07** | 0.03 | 1.00 | 0.01 | 1.04 | 0.04 | 1.03*** | 0.01 | 1.02** | 0.01 |
| Highest | 1.10** | 0.04 | 1.01 | 0.01 | 1.12** | 0.05 | 1.04*** | 0.01 | 1.04*** | 0.01 |

Note: *** p<0.01, ** p<0.05, * p<0.1

**Supplementary Table S6**: ATE and ATET estimates from PSM for comparison and intervention group from endline sample

| Domains | PSM Coefficient | |
| --- | --- | --- |
|  | ATE | ATT |
| Gender equitable roles | 4.03*** | 2.58*** |
|  | *(0.71)* | *(0.83)* |
| Diet and nutrition | 0.87*** | 0.76*** |
|  | *(0.10)* | *(0.13)* |
| Self-esteem | 0.18 | -0.18 |
|  | *(0.22)* | *(0.26)* |
| Self-efficacy | 0.42* | 0.16 |
|  | *(0.25)* | *(0.29)* |
| Employee rights and responsibilities | 0.62*** | 0.47*** |
|  | *(0.12)* | *(0.15)* |
| N | 1658 | 1658 |

Note: ***, ** and * denotes p-value significance at 1%, 5% and 10%, respectively.

Standard error of the PSM estimates is reported in parenthesis *(.)*.
